# ZEB1 directly activates IL-6 to promote triple-negative breast cancer stemness in an epigenetically constrained context

**DOI:** 10.3389/fonc.2026.1929305

**Published:** 2026-09-01

**Authors:** Weiyue Zhang, Yifan Zhou, Wei Ma, Huimin Jiang

**Affiliations:** 1 School of Biological Science and Medical Engineering, Beihang University, Beijing, China; 2 Beijing Institute of Brain Disorders, Laboratory of Brain Disorders, Ministry of Science and Technology, Collaborative Innovation Center for Brain Disorders, Beijing Advanced Innovation Center for Big Data-Based Precision Medicine, Capital Medical University, Beijing, China

**Keywords:** triple-negative breast cancer, cancer stem cells, ZEB1, IL-6, epigenetic regulation

## Abstract

ZEB1 is a key driver of breast cancer pathogenesis and therapy resistance, largely through its ability to promote cancer stem cells (CSCs). Although tumor-secreted cytokines such as IL-6 are known to support tumor progression, how their expression is regulated by upstream transcriptional factors such as ZEB1 remains incompletely understood. Here, we identify IL-6 as a direct transcriptional target of ZEB1 and reveal an epigenetic dimension associated with this regulation. Mechanistically, ZEB1 binds to specific E2-box elements within the IL-6 promoter and activates its transcription. The IL-6 promoter exhibits local DNA methylation, and ZEB1-dependent IL-6 expression was further enhanced by DNA methyltransferase and histone deacetylase inhibitors, suggesting that the local epigenetic context constrains IL-6 transcription. ZEB1 overexpression was also accompanied by enrichment of the active histone marks H3K27ac and H3K4me3 at the IL-6 promoter, consistent with a more transcriptionally permissive local chromatin state. Functionally, the ZEB1-induced IL-6 expression enhanced CSC-associated properties *in vitro* and tumor-initiating capacity *in vivo*. Conversely, IL-6 knockdown attenuated ZEB1-induced CSC-associated phenotypes, whereas pharmacological IL-6 neutralization reduced xenograft burden and CSC-associated populations *in vivo*. Collectively, our findings establish the ZEB1–IL-6 axis as an important regulator of triple-negative breast cancer (TNBC) stemness and indicate that ZEB1-dependent IL-6 activation is influenced by the local epigenetic context. These findings support further investigation of the ZEB1–IL-6 axis as a potential therapeutic target in ZEB1-high TNBC.

## Introduction

CSCs have recently garnered significant attention owing to their role in promoting tumor growth and inducing therapeutic resistance (1). Although they are scarce in the tumor microenvironment, CSCs exhibit unique self-renewal properties and therapeutic resistance, positioning them as critical mediators of carcinogenesis and metastatic recurrence. Therefore, targeting CSCs presents a promising strategy to overcome therapeutic resistance and improve treatment outcomes (2). Tumor cells are known to secrete various pro-inflammatory cytokines, chemokines, and other soluble factors into the tumor microenvironment. Previous studies have established that IL-6 promotes breast CSC maintenance, facilitates the dynamic conversion of non-CSCs into CSCs, and contributes to therapeutic resistance by expanding CSC populations. Thus, the pro-stemness function of IL-6 in breast cancer is well established. However, the upstream transcriptional mechanisms that sustain tumor-cell-derived IL-6 expression, particularly in TNBC, remain incompletely understood (3). Breast cancer can be classified into Luminal A, Luminal B, HER2 overexpression and TNBC according to molecular typing (4, 5). Among these, TNBC is associated with the most unfavorable prognosis, largely because CSCs are a major driver of chemotherapy and targeted therapy resistance (6, 7). Therefore, defining the tumor-cell-derived autocrine factors that sustain CSC properties is particularly important for understanding therapeutic resistance and tumor progression in TNBC.

In addition to its role in cell fate determination, ZEB1 functions as a molecular rheostat for stem cell plasticity, thereby influencing cellular behavior and tissue development (8–10). Analysis of TCGA datasets for breast cancer showed that ZEB1 amplification was an independent prognostic factor (11). Furthermore, the stemness-promoting activity of ZEB1 has been shown to be significantly associated with early metastatic recurrence in triple-negative cohorts (12). ZEB1 was found to transform non-invasive basal-type cancer cells into CSCs with enhanced invasiveness and metastatic potential. This process is mediated by a bivalent chromatin state at the ZEB1 locus, which permits rapid gene activation in response to signals like TGF-β. Subsequently, ZEB1 drives EMT and confers stem-like properties to CSCs (8, 9). Moreover, ZEB1 enhances radio-resistance in breast cancer by regulating the DNA damage repair pathway—a function closely linked to stemness (9). ZEB1 also maintains the stemness of cells by influencing key signaling pathways and metabolic reprogramming (13, 14). In summary, ZEB1 promotes breast cancer stemness through multiple interconnected pathways and is particularly relevant to the aggressive phenotype of TNBC. However, how ZEB1 regulates downstream pro-inflammatory cytokines to sustain TNBC stemness remains incompletely understood. While previous studies have established a link between ZEB1 and an inflammatory phenotype (15) and recently delineated the SIX1-ZEB1-IL-6 axis (16), whether ZEB1 directly regulates IL-6 transcription and how the local epigenetic context influences this regulation remain incompletely understood. Through transcriptomic analysis of ZEB1-overexpressing MDA-MB-231 cells, we identified multiple altered cytokines and prioritized IL-6 for further investigation based on its established role in CSC regulation and its therapeutic tractability. Accordingly, we sought to determine whether IL-6 functions as a downstream mediator of ZEB1-induced CSC-associated phenotypes in TNBC and to characterize the transcriptional basis and local epigenetic context of this regulation. Notably, knockdown of IL-6 significantly impaired ZEB1-induced stemness-associated phenotypes in TNBC cells. We identify ZEB1 as a direct transcriptional activator of IL-6 and characterize the local epigenetic context associated with this regulation. Through transcriptomic and mechanistic analyses, we demonstrate that ZEB1 binds to E2-box elements in the IL-6 promoter and promotes its transcription despite the presence of local DNA methylation. Notably, this activation is potentiated by pharmacological inhibition of DNA methyltransferases and histone deacetylases, suggesting that local epigenetic repression constrains ZEB1-dependent IL-6 activation. Our findings identify the ZEB1-IL-6 axis as an important regulator of TNBC stemness and support further investigation of this axis as a potential therapeutic target in ZEB1-high TNBC.

## Materials and methods

### Cell culture

The MDA-MB-231 cells were cultured in RPMI-1640 medium, while the SUM-159 cells were cultured in DMEM medium, both of which were supplemented with 10% FBS and 1% pen/strep. HEK293T cells were cultured in DMEM medium containing 10% FBS, 1% sodium pyruvate, 1% non-essential amino acids (NEAA) and 2% L-glutamine. All cell lines were tested negative for mycoplasma contamination.

### RNA extraction and quantitative real-time PCR

Total RNA was isolated from cultured breast cancer cells using TRIzol reagent (Life Technologies). cDNA was synthesized using M-MLV Reverse Transcriptase (Takara). Quantitative real-time PCR was performed using the TransStart Green Q-PCR SuperMix Kit (TransGen) to analyze the expression of IL-6. GAPDH was used as the endogenous control. Primer sequences are listed in **Supplementary Table 1**.

### Plasmid construction

The full-length human Zeb1 and IL-6 cDNAs were PCR-amplified and cloned into the pLV-EF1-MCS-IRES-Bsd lentiviral vector (Biosettia) for subsequent applications. The pLV-H1-EF1α-puro lentiviral vector (Biosettia) was then used to deliver shRNAs into breast cancer cells to mediate RNA interference-based gene knockdown. The human IL-6 full-length and truncated promoter sequences were obtained by PCR from human genomic DNA and cloned into the pGL3-basic vector. The primer sequences are shown in **Supplementary Table 1**.

### Generation of lentiviruses

Lentiviral particles were produced by calcium phosphate transfection of subconfluent HEK293T cells using the lentiviral transfer vector and packaging plasmids. The viral supernatant was harvested 48 hours after transfection, concentrated by ultracentrifugation at 75,000×g for 90 minutes, and then resuspended and filtered through 0.45 µm filters (Millipore).

### RNA-sequencing

ZEB1/231 cells and Ctrl/231 cells were first lysed with TRIzol reagent and further extracted following the manufacturer’s guidelines. Following extraction and DNase I treatment of total RNA, mRNA is isolated using Oligo(dT). This mRNA is then fragmented by mixing with fragmentation buffer, and cDNA is subsequently synthesized using the fragmented mRNA as templates. Short fragments are purified and resolved with EB buffer for end reparation and single nucleotide A (adenine) addition. After that, the short fragments are connected with adapters. The suitable fragments are selected for the PCR amplification. During the QC steps, Agilent 2100 Bioanaylzer and ABI Step OnePlus Real-Time PCR System are used in quantification and qualification of the sample library. Then the library is sequenced using Illumina HiSeq 4000. To observe phenotypic change, we adopted differential genes that had been annotated to carry out KEGG (https://www.kegg.jp/kegg/kegg1.html) enrichment analysis by Phyper based on the Hypergeometric test.

### Animals

BALB/c nude mice (female, 6–8 weeks old, weight 18–22 g) were purchased from Beijing Vital River Laboratory Animal Technology Co., Ltd. (Beijing, China). Mice were specific-pathogen-free (SPF) and housed under standard conditions (24 °C, 55% humidity, 12-h light/dark cycle) in ventilated cages. All procedures were approved by the Capital Medical University Institutional Animal Care and Use Committee (Approval No. AEEI-2025-200) and conducted in accordance with relevant guidelines. In addition, the study was performed in accordance with ARRIVE 2.0 guidelines.

### Tumor cell implantation

Female BALB/c nude mice aged 6 to 8 weeks were randomly assigned to each group. MDA-MB-231 cells were trypsinized to generate single-cell suspension, resuspended in PBS, counted, diluted to the desired concentration, and orthotopically injected into the fourth mammary fat pads in BALB/c nude mice. Tumor dimensions were measured three times at each assessment, and the mean values were recorded. Animals were euthanized if tumors exceeded 1,500 mm³ or showed signs of ulceration/distress. At the end of the experiment, all mice were euthanized with 100 mg/kg of pentobarbital sodium, and then the tumor tissues were completely removed, weighed, and archived for photography.

For the *in vivo* limiting-dilution assay, Ctrl/231 or IL-6/231 cells were injected into the mammary fat pads of nude mice at doses of 1.0 × 10^6^, 5.0 × 10^5^, 2.5 × 10^5^, 1.25 × 10^5^, or 6.25 × 10^4^ cells per injection. Six mice were used per group at each cell dose. Xenografts were harvested approximately 4 weeks after cell inoculation.

For IL-6 neutralization, when the xenograft tumors reached approximately 50 mm³, mice were randomly assigned to receive either an IL-6-neutralizing antibody or the corresponding IgG control (17). The IL-6-neutralizing antibody or IgG was administered peritumorally at a dose of 100 μg per mouse in 50 μL, twice weekly for 2 consecutive weeks. Tumor volume was measured every 3 days using digital calipers and calculated as V = 0.5 × length × width². Tumor weight was determined at the experimental endpoint.

### ELISA assay

Conditioned culture supernatants were collected from the indicated cell groups at 48 h after the medium was replaced. Cellular debris was removed by centrifugation, and the supernatants were stored at −80 °C until analysis. IL-6 concentrations were measured using a human IL-6 ELISA kit according to the manufacturer’s instructions.

### Immunoblotting assay

Protein extracts were obtained through cell lysis in RIPA buffer freshly supplemented with protease inhibitor and phosphatase inhibitors. Protein concentrations were determined using a BCA assay. Proteins were separated on 10% SDS-PAGE gels, with 30 μg of protein loaded per lane. Following electrophoretic transfer to polyvinylidene fluoride (PVDF) membranes, the membranes were first blocked with 5% skim milk for 1 hour at room temperature, then incubated overnight at 4 °C with primary antibodies (IL-6, 21865-1-AP, Proteintech, 1:1000; β-actin, sc-47778, Santa Cruz, 1:1000), followed by a 1- hour incubation with species-matched secondary antibodies at room temperature. Protein signals were detected using an enhanced chemiluminescence (ECL) kit and captured with a chemiluminescence imaging system. Band intensities were quantified using ImageJ software. IL-6 band intensity was normalized to the corresponding β-actin signal, and the results were expressed relative to the control group.

### Flow cytometry

For xenograft-derived samples, fresh tumor tissues were minced and enzymatically dissociated into single-cell suspensions using collagenase at 37 °C for 30 min. The dissociated cells were passed through a 40-μm cell strainer and washed with ice-cold PBS containing 2% FBS. Human EpCAM-positive tumor cells were subsequently enriched using magnetic beads according to the manufacturer’s instructions. The EpCAM-enriched tumor-cell fraction was used for subsequent CD44/CD24 staining and ALDEFLUOR analysis.

For cell surface marker profiling, cells were resuspended in ice-cold PBS supplemented with 2% FBS, followed by incubation with fluorochrome-conjugated anti-human CD44 (BD Biosciences, 559942) and CD24 (BD Biosciences, 555428) antibodies for 30 minutes at 4 °C in the dark. Unbound antibodies were removed by three washes with PBS-2% FBS buffer prior to flow cytometry analysis. Unstained cells and single-stained controls, including CD24-PE-only and CD44-APC-only samples, were included for fluorescence compensation and gate setting. Gates were defined using these controls and were applied consistently to all samples within the same experiment.

ALDH enzymatic activity was assessed using the ALDEFLUOR™ Kit (Stemcell Technologies, 01700) following the manufacturer’s standardized protocol. Briefly, single-cell suspensions were incubated with the ALDH substrate BODIPY- BAAA at 37 °C for 45 minutes in the dark. For each experimental group, an aliquot of cells was treated in parallel with the ALDH inhibitor diethylaminobenzaldehyde (DEAB) as a matched negative control. The ALDH-positive gate for each group was defined relative to its corresponding DEAB-treated control. Cells were washed twice with assay buffer and analyzed by flow cytometry within 1 h. Samples compared within the same experiment were cultured, stained, acquired, and analyzed in parallel using identical reagent concentrations, instrument settings, and gating criteria.

For side population (SP) analysis, cells were resuspended in 37 °C PBS containing 2% FBS at a density of 1×10^6^ cells/ml. Cells were incubated with 5 μg/ml Hoechst 33342 in the presence or absence of 50 μM verapamil for 1.5 h at 37 °C in darkness with intermittent shaking. The SP gate was defined as the Hoechst-low population that was markedly reduced in the presence of verapamil, which was used as an inhibitor control for Hoechst 33342 efflux. All experiments using *in vitro* cells were performed in at least three independent experiments.

### Methylation-specific PCR

Genomic DNA was extracted from breast cancer cells using a commercial DNA extraction method and subjected to bisulfite conversion. The methylation status of CpG sites within the IL-6 promoter was analyzed by methylation-specific PCR (MSP). Primer sequences are listed in **Supplementary Table 1**.

### Luciferase assay

Following the transfection of constructs containing wild-type or mutant human IL-6 promoters into 24-well plates, the cell lysates were harvested 36 hours after transfection. Luciferase activity was then quantified using the Dual-Luciferase Reporter Assay System (Promega), in accordance with the manufacturer’s instructions. Firefly luciferase activity was normalized to the activity of the co-transfected Renilla luciferase, which served as an internal control.

### Chromatin immunoprecipitation (ChIP)

ChIP assays were performed using the EZ-ChIP kit (Millipore) according to the manufacturer’s instructions. Cells were crosslinked with 1% formaldehyde for 10 min at room temperature, followed by quenching with 125 mM glycine. Chromatin extracts containing DNA fragments were immunoprecipitated using specific antibodies. The ChIP-enriched DNA was then uncrosslinked and subjected to quantitative PCR. The primers used in these experiments are shown in **Supplementary Table 1**.

### Statistical analysis

Statistical analyses were performed using GraphPad Prism 8 software. All data are presented as the mean ± SEM, representing three or more independent experiments. One-way analysis of variance (ANOVA) was used to compare the means of the different treatment groups. Where appropriate, a student’s t-test for unpaired observations was applied. Longitudinal tumor-volume data were analyzed using two-way repeated-measures ANOVA with the Geisser–Greenhouse correction. A p-value of less than 0.05 was considered significant.

## Results

### IL-6 promotes stemness-associated properties in TNBC cells

While ZEB1 is known to play an important role in tumor initiation and development, it remains unclear whether it promotes breast cancer cell stemness by regulating autocrine mechanisms (9, 15, 18). To identify secreted cytokines downstream of ZEB1, we performed RNA sequencing on empty vector-expressing (Ctrl/231) and ZEB1-overexpressing (ZEB1/231) MDA-MB-231 cells. KEGG pathway analysis revealed that ZEB1 overexpression led to significant enrichment of genes involved in cytokine-cytokine receptor interaction and chemokine signaling pathways (**Supplementary Figure 1A**). The heatmap depicting differentially expressed cytokines and chemokines in Ctrl/231 versus ZEB1/231 cells revealed a spectrum of alterations (**Supplementary Figure 1B**). Although IL-6 was not the most strongly altered cytokine in the RNA-sequencing dataset, it was prioritized for mechanistic investigation based on its well-established role in regulating CSC maintenance and plasticity, particularly in breast cancer, as well as the availability of clinically relevant strategies targeting IL-6 signaling. Thus, IL-6 was selected on the basis of its biological relevance and translational tractability rather than solely on the magnitude of its expression change. Subsequent validation showed that ZEB1 overexpression consistently increased IL-6 expression at both the mRNA and protein levels in MDA-MB-231 and SUM-159 cells (**Supplementary Figures 1C–F**), whereas ZEB1 knockdown reduced IL-6 expression (**Supplementary Figures 1G–J**), further supporting IL-6 as a relevant downstream effector of ZEB1.

**Figure 1:**
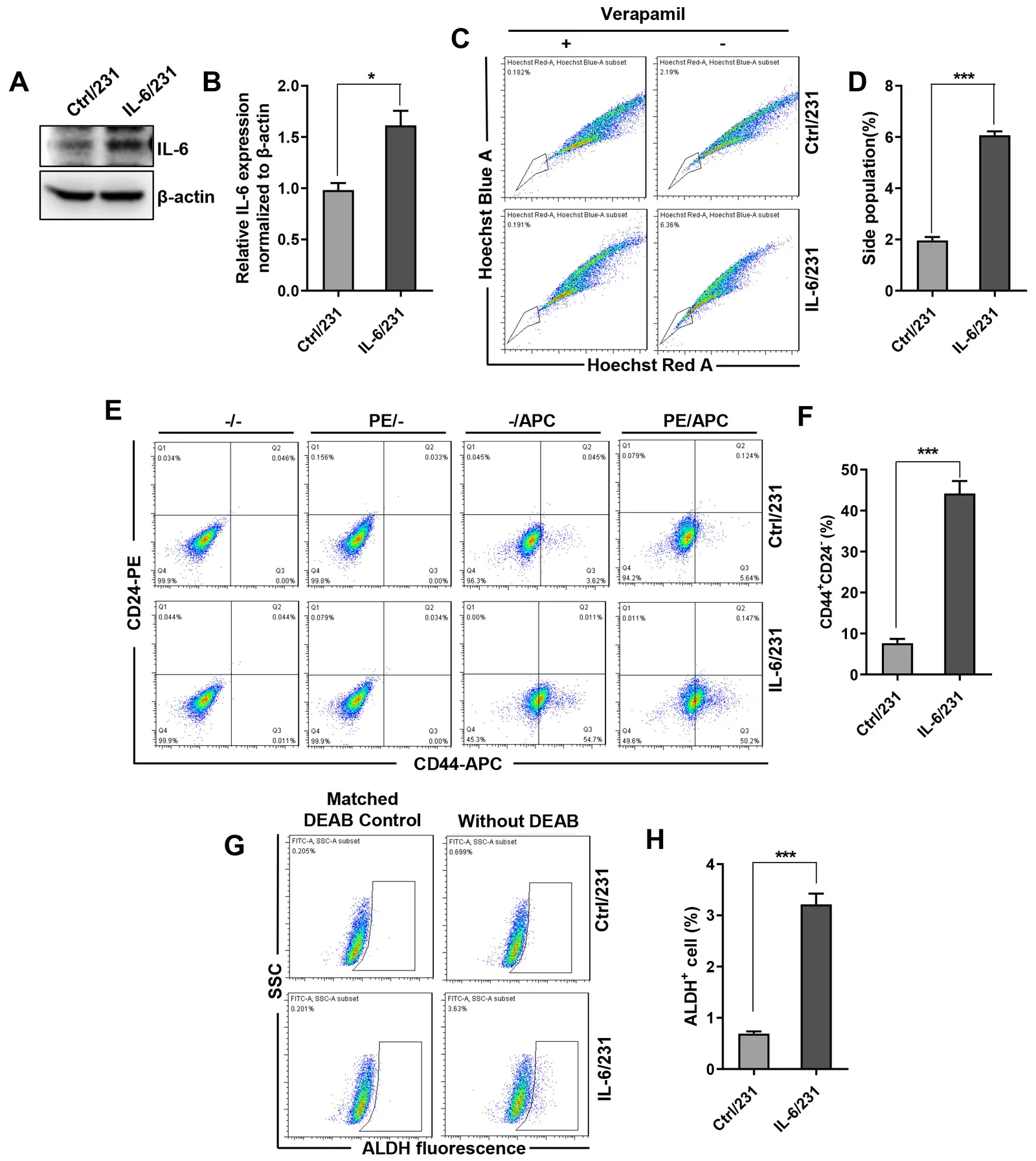
IL-6 enhances CSC-associated properties in MDA-MB-231 cells. **(A)** Representative immunoblot showing IL-6 expression in MDA-MB-231 cells stably transfected with an IL-6 expression plasmid (IL-6/231) or empty vector control (Ctrl/231). **(B)** Relative IL-6 protein levels normalized to β-actin and expressed relative to the Ctrl/231 group. C-H. Flow cytometry analysis of the side-population assay **(C, D)**, the CD44^+^CD24^−^ population **(E, F)**, and ALDH activity **(G, H)** in Ctrl/231 and IL-6/231 cells. Verapamil-treated cells were used to define the SP gate. In the plots, −/−, PE/−, −/APC, and PE/APC denote unstained, CD24-PE single-stained, CD44-APC single-stained, and CD24-PE/CD44-APC double-stained samples, respectively. n = 3 independent experiments. Data are represented as the mean ± SEM. *P < 0. 05, ***P < 0. 001.

As a prerequisite for examining IL-6 as a downstream mediator of ZEB1, we first validated the effects of IL-6 on CSC-associated phenotypes in our TNBC models. To this end, we overexpressed IL-6 in MDA-MB-231 cells (**Figures 1A, B**). IL-6 overexpression resulted in an expansion of the side population (SP) in MDA-MB-231 cells (**Figures 1C, D**). The SP phenotype reflects increased Hoechst 33342 efflux activity and is commonly used as a functional readout enriched for stem-like and drug-resistant tumor cells (19, 20). The SP gate was verified using verapamil-treated cells. Furthermore, flow cytometry analysis demonstrated that IL-6 overexpression enriched multiple stemness-associated metrics, namely the CD44^+^CD24^−^ population (**Figures 1E, F**) and ALDH activity (**Figures 1G, H**), compared to control cells. MDA-MB-231 cells displayed predominantly CD44-positive/CD24-negative staining, consistent with the previously reported phenotype of this cell line (21, 22). No appreciable CD24-positive population was detected under the staining and gating conditions used in the present study; therefore, the analysis focused on changes in the CD44^+^/CD24^-^ population. We validated these findings in SUM-159 cells, where IL-6 similarly enhanced CSC-associated properties (**Supplementary Figure 2**), further supporting a consistent role for IL-6 in promoting CSC-associated phenotypes in the two TNBC cell models examined.

### IL-6 enhances tumor-initiating capacity and CSC enrichment in TNBC xenografts

**Figure 2:**
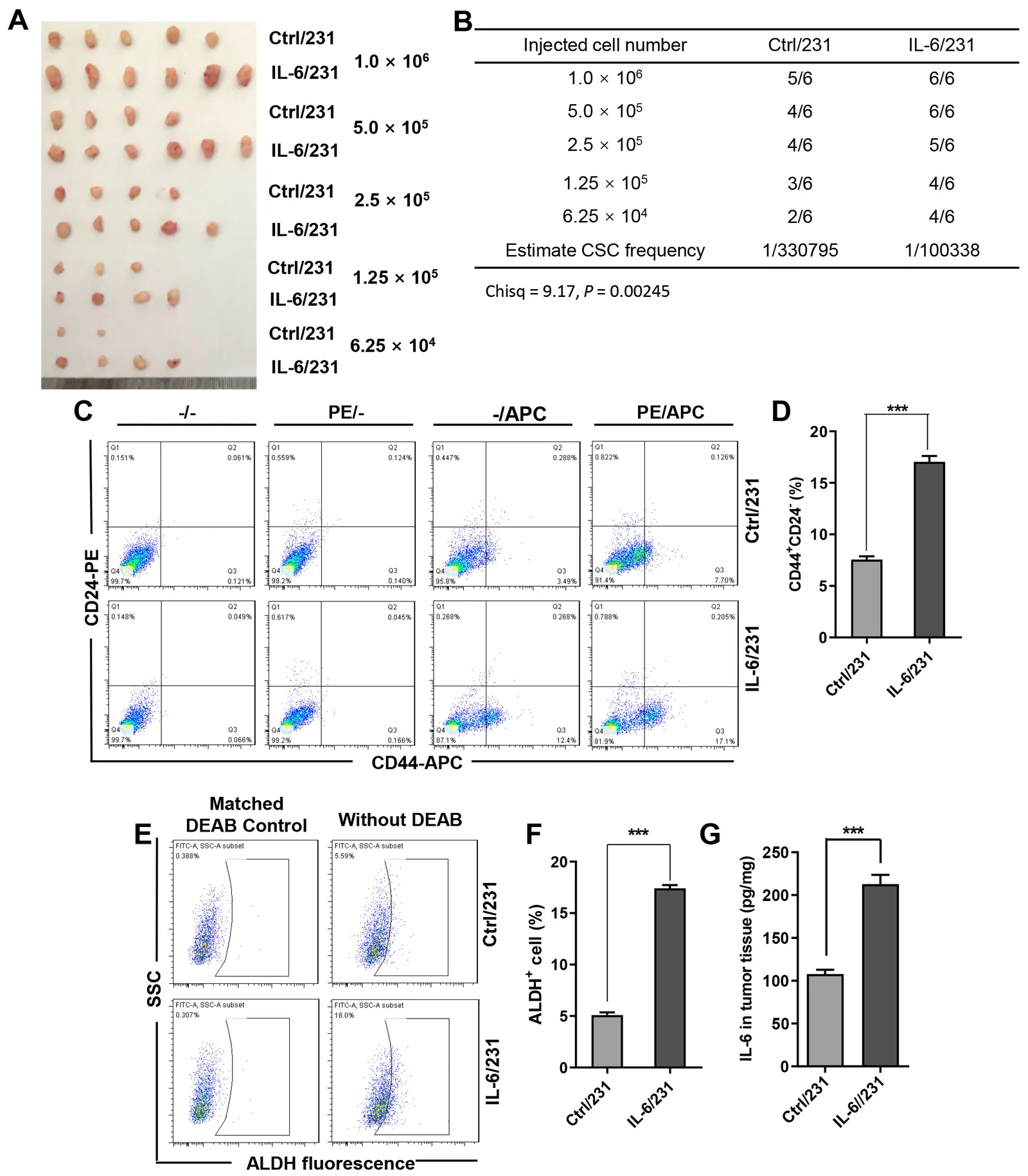
IL-6 increases tumor-initiating capacity and CSC-associated properties in MDA-MB-231 xenografts. **(A)** For *in vivo* limiting-dilution assays, 1×10^6^, 5×10^5^, 2.5×10^5^, 1.25×10^5^, and 6.25×10^4^ IL-6/231 or Ctrl/231 cells were injected into the mammary fat pads of nude mice. Tumors from mice in each group were shown, n = 6 mice per group at each cell dose. **(B)** The estimated CSC frequency was analyzed using ELDA software. *P* = 0.00245. **(C–F)**. Flow cytometry analysis of the CD44^+^CD24^−^ population **(C, D)**, and ALDH activity **(E, F)** in human EpCAM-enriched tumor cells isolated from dissociated xenografts, n = 3 per group. **(G)** Quantification of IL-6 by ELISA in Ctrl/231 and IL-6/231-derived xenograft tumors, n = 5 per group. Data are represented as the mean ± SEM. ***P < 0. 001.

We next examined whether the IL-6-induced CSC-associated phenotypes observed *in vitro* were accompanied by increased tumor-initiating capacity *in vivo*. Ctrl/231 and IL-6/231 cells were transplanted into the mammary fat pads of BALB/C nude mice. Limiting-dilution analysis demonstrated that IL-6 overexpression significantly increased the estimated tumor-initiating cell frequency from 1/330,795 in the Ctrl/231 group to 1/100,338 in the IL-6/231 group (**Figures 2A, B**). Flow cytometric analysis of human EpCAM-enriched tumor cells isolated from the xenografts further demonstrated that IL-6 overexpression increased the CD44^+^CD24^-^ population and ALDH activity (**Figures 2C–F**), consistent with enrichment of CSC-associated populations. ELISA analysis of homogenates prepared from the primary xenograft tumors confirmed that IL-6/231-derived tumors contained significantly higher levels of IL-6 protein than Ctrl/231-derived tumors (**Figure 2G**). These findings indicate that IL-6 overexpression enhances tumor-initiating capacity and enriches CSC-associated populations in the MDA-MB-231 xenograft model.

### Targeting IL-6 attenuates stemness-associated properties in TNBC cells

**Figure 3:**
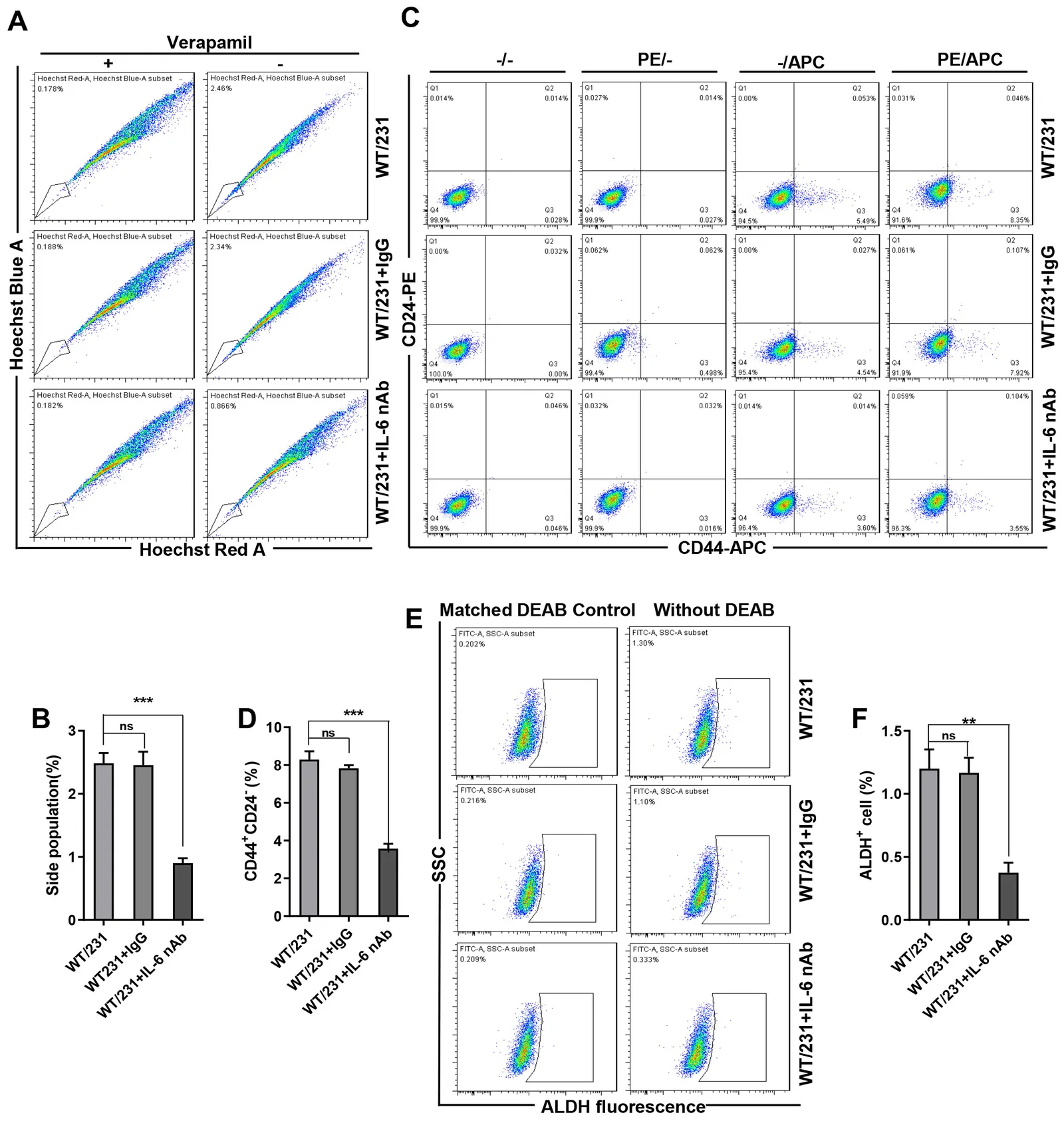
Targeting IL-6 attenuates CSC-associated properties in MDA-MB-231 cells. **(A–F)** Flow-cytometric analysis of the side-population fraction **(A, B)**, the CD44^+^CD24^−^ population **(C, D)**, and ALDH activity **(E, F)** in untreated MDA-MB-231 cells and cells treated with control IgG or IL-6 neutralizing antibody. For the ALDEFLUOR assay, a matched DEAB-treated negative control was included for each experimental group and was used to define the corresponding ALDH-positive gate. n = 3 independent experiments. Data are represented as the mean ± SEM. **P< 0. 01, ***P < 0. 001.

To complement the gain-of-function experiments and confirm that endogenous IL-6 signaling supports CSC-associated phenotypes in our TNBC model, we next examined the effects of IL-6 blockade. To this end, MDA-MB-231 cells were treated with an IL-6-neutralizing antibody. We assessed the functional impact of this blockade on well-established stemness-associated phenotypes. The results demonstrated that IL-6 inhibition significantly reduced the side population (SP) fraction (**Figures 3A, B**) and diminished the abundance of the CD44^+^CD24^-^ cell subpopulation (**Figures 3C, D**). Crucially, IL-6 blockade also reduced the ALDH-positive fraction, as determined using the ALDEFLUOR assay with a matched DEAB-treated control for each experimental group (**Figures 3E, F**). All groups within this independent experiment were processed and analyzed in parallel under identical conditions. These results confirmed that IL-6 blockade attenuates multiple CSC-associated phenotypes in MDA-MB-231 cells.

### Targeting IL-6 reduces TNBC xenograft burden *in vivo*

**Figure 4:**
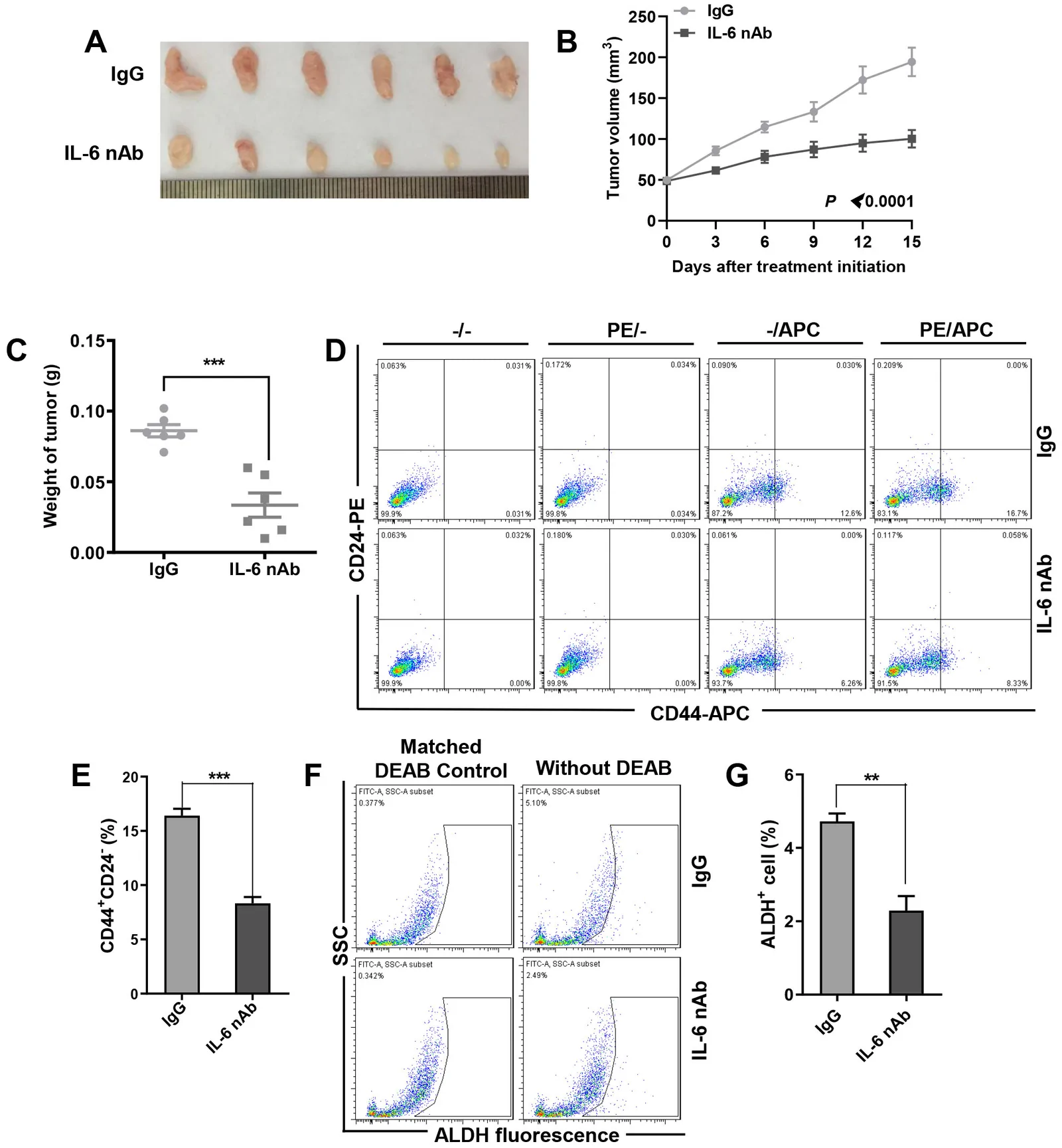
IL-6 neutralization reduces xenograft burden and CSC-associated populations in MDA-MB-231 xenografts. **(A)** A total of 1 × 10^6^ MDA-MB-231 cells were injected into the mammary fat pads of nude mice to allow the establishment of primary tumors, followed by treatment with IgG and IL-6 neutralizing antibody, n = 6 mice per group. **(B)** Tumor-volume curves. Longitudinal tumor-volume data were analyzed using two-way repeated-measures ANOVA with the Geisser–Greenhouse correction; the treatment-by-time interaction was significant (*P* < 0.0001). **(C)** Tumor weights at the experimental endpoint, n = 6 mice per group. **(D–G)** Flow cytometry analysis of the CD44^+^CD24^−^ population **(D, E)**, and ALDH activity **(F, G)** in EpCAM-enriched tumor cells isolated from dissociated xenografts. Matched DEAB-treated controls were used to define the ALDH-positive gates, n = 3 per group. Data are represented as the mean ± SEM. **P < 0. 01, ***P< 0. 001.

We next examined whether pharmacological neutralization of IL-6 affected xenograft burden and CSC-associated populations in the MDA-MB-231 xenograft model. IL-6 neutralization significantly reduced xenograft volume over time, as demonstrated by a significant treatment-by-time interaction (*P* < 0.0001, two-way repeated-measures ANOVA with the Geisser–Greenhouse correction; **Figures 4A, B**). Final tumor weight was also significantly reduced in the IL-6-neutralizing antibody group compared with the IgG control group (**Figure 4C**). To assess CSC-associated populations, resected xenografts were enzymatically dissociated into single-cell suspensions, followed by magnetic enrichment of human EpCAM-positive tumor cells before flow-cytometric analysis. Analysis of the EpCAM-enriched tumor-cell fraction showed that IL-6 neutralization significantly reduced the CD44^+^CD24^-^ population (*P* = 0.0007) and the ALDH-positive population (*P* = 0.0059) compared with the IgG control group (**Figures 4D–G**). Collectively, these results indicate that IL-6 neutralization reduces MDA-MB-231 xenograft burden and decreases CSC-associated populations *in vivo*.

### IL-6 knockdown attenuates ZEB1-induced CSC-associated properties in TNBC cells

**Figure 5:**
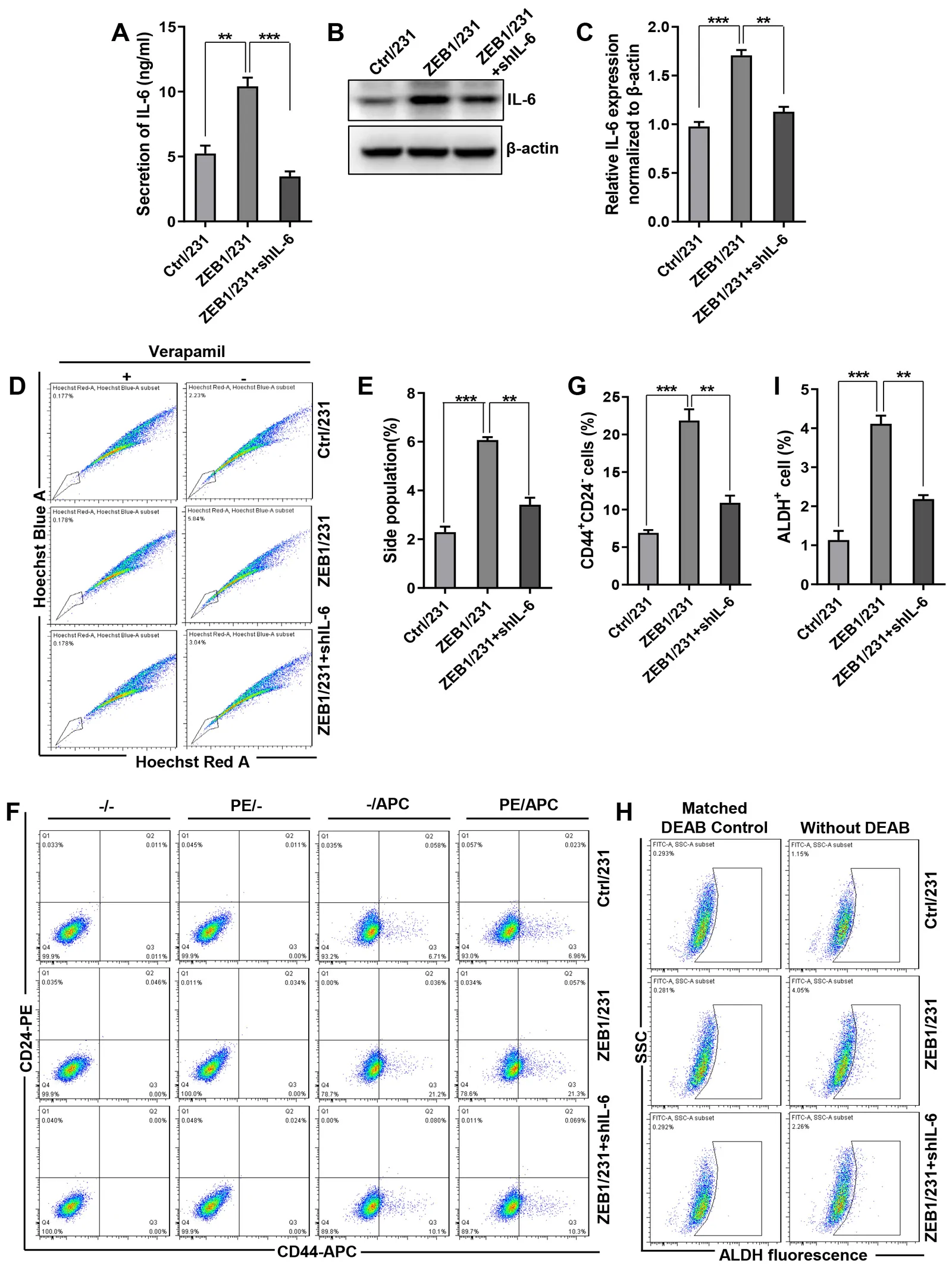
IL-6 is an important mediator of ZEB1-induced CSC-associated properties in TNBC cells. **(A)** IL-6 concentrations in conditioned culture supernatants from Ctrl/231, ZEB1/231, and ZEB1/231 + shIL-6 cells were measured by ELISA. **(B)** Representative immunoblot showing IL-6 expression in the indicated groups. **(C)** Quantification of IL-6 protein expression normalized to β-actin. D-I. Flow cytometry analysis of the side-population **(D, E)**, the CD44^+^CD24^−^ population **(F, G)**, and the ALDH activity **(H, I)** for Ctrl/231, ZEB1/231 and ZEB1/231+shIL-6 cells. n = 3 independent experiments. Data are represented as the mean ± SEM. **P < 0. 01, ***P < 0. 001.

To directly determine whether IL-6 functions as a downstream mediator of ZEB1-induced CSC-associated phenotypes, shRNA-mediated IL-6 knockdown was introduced into ZEB1-overexpressing MDA-MB-231 cells. ELISA analysis of conditioned culture supernatants showed that ZEB1 overexpression significantly increased IL-6 secretion, whereas IL-6 knockdown markedly attenuated this increase (**Figure 5A**). Immunoblotting and quantitative analysis further confirmed the corresponding changes in cellular IL-6 protein expression (**Figures 5B, C**). Functionally, the ZEB1-induced increases in the SP fraction (**Figures 5D, E**), the CD44^+^CD24^-^ population (**Figures 5F, G**), and ALDH activity (**Figures 5H, I**) were significantly attenuated by IL-6 knockdown. These findings were consistently recapitulated in SUM-159 cells, where IL-6 knockdown similarly attenuated ZEB1-induced CSC-associated phenotypes (**Supplementary Figure 3**). Collectively, these loss-of-function experiments identify IL-6 as an important downstream mediator of ZEB1-induced CSC-associated phenotypes in TNBC cells.

### ZEB1 directly activates IL-6 transcription in an epigenetically constrained context

**Figure 6:**
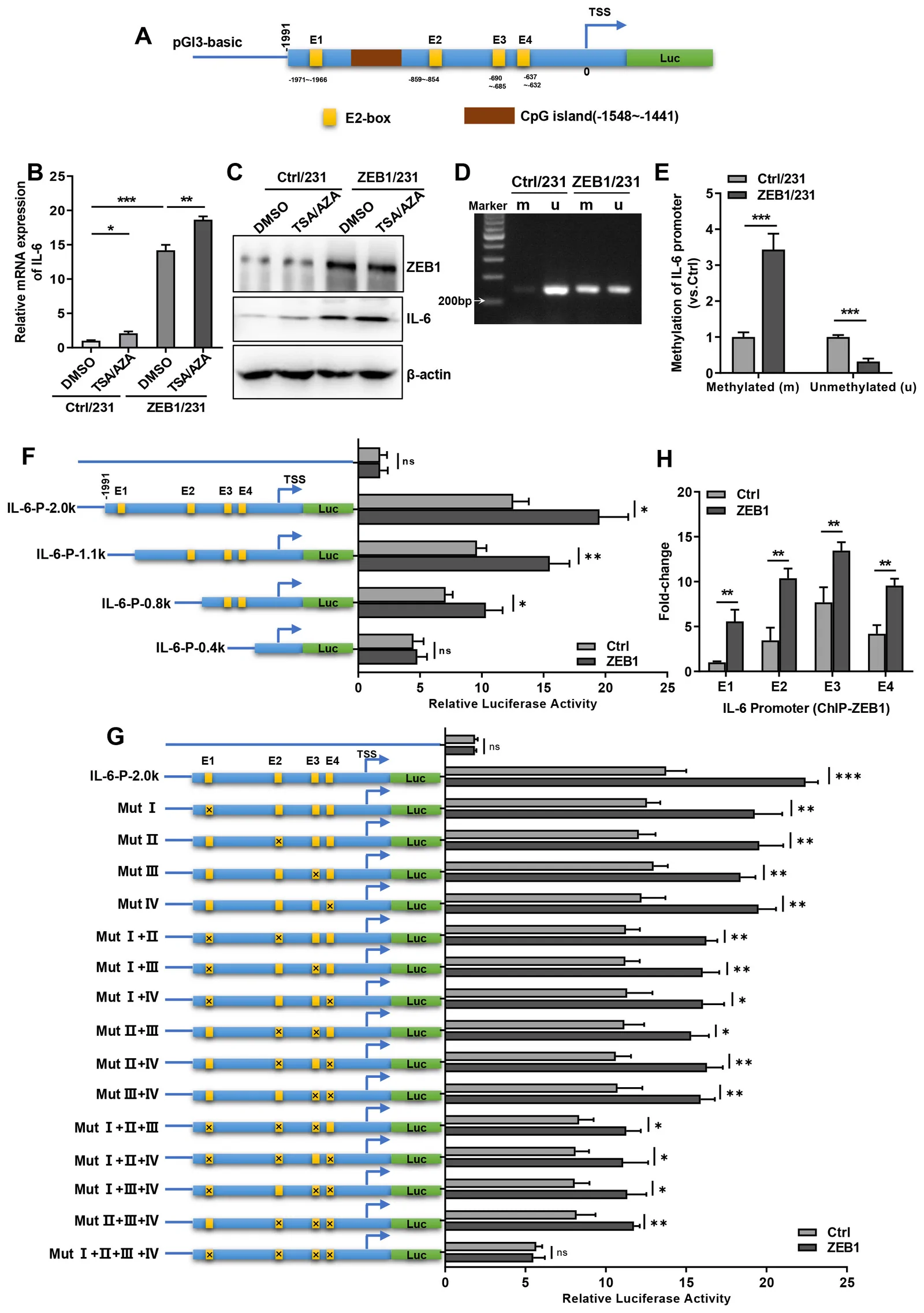
ZEB1 directly activates the IL-6 promoter in an epigenetically constrained context. **(A)** An upstream CpG-rich region was located at position −1548/−1441 of the IL-6 promoter, and four canonical E2-box elements for ZEB1 binding were identified within. **(B, C)** IL-6 mRNA expression **(B)** and representative immunoblots of IL-6 and ZEB1 protein expression **(C)** in Ctrl/231 and ZEB1/231 cells following DMSO or AZA/TSA treatment. **(D, E)** The methylation status of the IL-6 promoter was assessed by MSP in ZEB1/231 versus Ctrl/231 cells. **(F, G)** Luciferase assays for wild-type (−1991/+51), truncated **(F)** or site-directed mutagenesis **(G)** promoters of IL-6 in Ctrl/231 and ZEB1/231 cells. **(H)** ChIP assay for recruitment of ZEB1 to the endogenous IL-6 promoter in Ctrl/231 and ZEB1/231 cells. n = 3 independent experiments. Data are represented as the mean ± SEM. *P < 0. 05, **P < 0. 01, ***P < 0. 001.

Emerging evidence suggests that ZEB1 plays a pivotal role in epigenetic remodeling during cancer progression (23). To investigate whether the local epigenetic context influences ZEB1-dependent IL-6 regulation in TNBC cells, we first analyzed the IL-6 promoter region. Using MethPrimer, we identified a CpG island spanning positions -1548 to -1441 upstream of the transcription start site (**Figure 6A**), indicating its potential for methylation-mediated control. Given that promoter methylation often involves the coordinated action of DNA methyltransferases (DNMTs) and histone deacetylases (HDACs) (24), we treated ZEB1-overexpressing MDA-MB-231 cells with 5-aza-2’-deoxycytidine (AZA, a DNA demethylating agent) and trichostatin A (TSA, an HDAC inhibitor). Combined AZA/TSA treatment increased IL-6 mRNA expression in control cells and further enhanced ZEB1-dependent IL-6 transcription (**Figure 6B**). Immunoblotting showed that AZA/TSA treatment did not significantly alter ZEB1 protein abundance but modestly increased IL-6 protein expression, which was further supported by quantitative analysis of protein levels normalized to β-actin (**Figure 6C**; **Supplementary Figures 4A, B**). These findings are consistent with basal epigenetic repression of the IL-6 promoter and indicate that relief of this repression potentiates ZEB1-dependent IL-6 activation. Interestingly, MSP analysis revealed that ZEB1 overexpression was associated with enhanced DNA methylation of the IL-6 promoter (**Figures 6D, E**). This unexpected association indicates that increased promoter methylation is unlikely to be the primary driver of ZEB1-dependent IL-6 activation. Instead, it may reflect a feedback response, locus heterogeneity, or a consequence of transcriptional regulation; however, the underlying mechanism remains to be determined. To determine whether ZEB1 directly activates IL-6 transcription, we characterized the IL-6 promoter. The full-length -1991/+51 promoter of the human IL-6 gene has four putative ZEB1 binding sites (E_2_-box) at positions -1971/-1966, -859/-854, -690/-685 and -637/-632 (**Figure 6F**). The luciferase assay indicated that ZEB1 overexpression increased the promoter activity of the IL-6-p-2.0k reporter by approximately 2-fold relative to that of the control (**Figure 6F**). A series of truncated IL-6 promoter-reporter constructs were then generated for analysis. The results showed that deletion or site-directed mutagenesis of either E1, E2, E3, or E4 did not affect ZEB1-induced transcriptional activation of the IL-6 promoter, whereas simultaneous deletion or mutation of all ZEB1 binding sites completely eliminated this effect (**Figures 6F, G**). Mechanistically, the quantitative ChIP results demonstrated that overexpression of ZEB1 significantly increased its recruitment to E1, E2, E3, and E4 elements in the IL-6 promoter, demonstrating a predominant role for E2-boxes in the activation of IL-6 transcription by ZEB1 (**Figure 6H**). To resolve the apparent paradox between ZEB1-induced DNA methylation and IL-6 activation, we further examined the chromatin state via ChIP-qPCR. Our results revealed that ZEB1 overexpression was accompanied by enrichment of the active histone marks H3K27ac and H3K4me3 at the IL-6 promoter, whereas H3K27me3 remained largely unchanged. Collectively, these data suggested that ZEB1 directly activates IL-6 transcription by binding to its promoter in an E_2_ box-dependent manner. Furthermore, the potentiation of IL-6 expression by AZA/TSA treatment indicates that local epigenetic repression constrains IL-6 transcription and that relief of this constraint enhances ZEB1-dependent activation.

## Discussion

Cancer stem cells (CSCs), despite constituting a minor subset within tumors, contribute substantially to therapeutic resistance through their capacities for self-renewal, differentiation, and tumor regeneration (25, 26). Accumulating evidence has shown that tumor-derived cytokines, particularly IL-6, play pivotal roles in sustaining CSC properties and driving tumor progression. Therefore, targeting tumor-derived autocrine cytokines represents a promising therapeutic strategy. In this context, our findings support the ZEB1-IL-6 axis as an important mechanism contributing to the maintenance of TNBC stemness and highlight an epigenetic dimension to this regulation. We demonstrate that ZEB1 acts as an upstream regulator by binding directly to the IL-6 promoter and activating its transcription at a locus that exhibits local DNA methylation. The potentiation of ZEB1-dependent IL-6 expression by DNA methyltransferase and histone deacetylase inhibitors suggests that the local epigenetic context influences IL-6 transcription. However, these experiments do not establish that ZEB1 itself directly remodels chromatin at this locus. Thus, our work delineates a direct ZEB1–IL-6 transcriptional pathway that contributes to the maintenance of CSC properties and is influenced by the local epigenetic context in TNBC.

High ZEB1 expression has been associated with unfavorable clinical outcomes, tumor progression, therapeutic resistance, and reduced survival in breast cancer (8, 12, 27). Functionally, ZEB1 can promote tumor progression and stemness through EMT-dependent and EMT-independent mechanisms. In this study, we identify IL-6 as a direct transcriptional target of ZEB1 that contributes to its stemness-promoting effects in TNBC cells. Although ZEB1 is classically recognized as a transcriptional repressor, its transcriptional activity is context dependent and is influenced by the cofactors recruited at individual target promoters. A previous study demonstrated that ZEB1 can form a transcriptional complex with the histone acetyltransferases p300 and PCAF and recruit these co-activators to the ATM promoter, thereby activating ATM transcription in breast cancer cells (28). This finding provides a mechanistic precedent for the ability of ZEB1 to function as a transcriptional activator. In the present study, promoter-reporter assays, E2-box mutagenesis, and ChIP analyses demonstrate that ZEB1 directly binds to and activates the IL-6 promoter. However, whether ZEB1 recruits p300, PCAF, or other transcriptional co-activators to the IL-6 promoter was not directly examined and remains to be determined.

Importantly, our chromatin immunoprecipitation analyses reveal that this activation is accompanied by enrichment of active histone marks, including H3K27ac and H3K4me3, at the IL-6 promoter, while the repressive mark H3K27me3 remains largely unchanged (**Supplementary Figures 4C–E**). Together, these results show that ZEB1-dependent IL-6 activation is associated with enrichment of active histone marks at the IL-6 promoter. Previous studies provided frameworks for understanding the ZEB1-IL-6 relationship, including correlations with inflammatory factors (15) and identification of ZEB1 binding sites (16). Building upon these findings, we demonstrate that ZEB1 binds to and activates the IL-6 promoter despite the presence of detectable local DNA methylation. We further demonstrate that shRNA-mediated IL-6 knockdown attenuates ZEB1-induced CSC-associated phenotypes in both MDA-MB-231 and SUM-159 cells, functionally supporting IL-6 as an important downstream mediator of ZEB1 in the TNBC models examined. In our study, ZEB1 overexpression not only transcriptionally activated IL-6 but also concomitantly increased DNA methylation at the IL-6 promoter CpG island. This methylation change may represent a feedback response or an epiphenomenon secondary to transcriptional regulation, rather than a direct causal event. The coexistence of active transcription and concurrent promoter hypermethylation points to a complex interplay between transcriptional and epigenetic mechanisms. Although the precise nature of this regulation remains to be elucidated, it is clear that ZEB1 upregulation enhances IL-6 expression. Further studies are warranted to dissect the relationship between DNA methylation and ZEB1-dependent IL-6 transcription, and to determine how these two layers of control jointly shape the final output.

Although IL-6 was not the most strongly altered cytokine following ZEB1 overexpression, it was prioritized because of its established role in CSC regulation and its therapeutic tractability. The functional experiments in the present study supported IL-6 as an important downstream mediator of ZEB1-induced stemness-associated phenotypes. Nevertheless, other ZEB1-responsive cytokines, including IL-37 and CXCL8, may also contribute to these effects and warrant further investigation.

Previous studies have established that IL-6 promotes breast CSC maintenance, facilitates the conversion of non-CSCs into CSCs, and contributes to therapeutic resistance through the expansion of CSC populations. Consistent with these reports, our gain- and loss-of-function experiments confirmed that IL-6 enhances CSC-associated phenotypes and tumor-initiating capacity in the TNBC models used in this study. These experiments provided the functional basis for investigating whether IL-6 mediates ZEB1-induced stemness.

The SP phenotype reflects Hoechst 33342 efflux activity and is commonly used as a functional readout enriched for stem-like and drug-resistant tumor cells. Because no single marker or assay can definitively identify CSCs, we interpreted the SP results together with CD44^+^/CD24^-^ profiling, ALDH activity, and *in vivo* tumor-initiating assays. The absolute frequencies of flow-cytometrically defined CD44^+^/CD24^-^ and ALDH-positive populations may vary depending on cell passage, culture density, growth phase, and analytical conditions. Accordingly, the ALDH-positive fractions obtained in **Figures 1** and **3**, which represent independent experimental series, were not interpreted through direct comparison of their absolute baseline percentages. Instead, our conclusions were based on reproducible relative differences between matched groups cultured, stained, acquired, and analyzed in parallel within each experiment. Consistent with the predominantly CD24-negative phenotype previously reported for MDA-MB-231 cells, no appreciable CD24-positive population was detected under our experimental conditions.

The experimental scope of the present study was restricted to the TNBC cell lines MDA-MB-231 and SUM-159; therefore, whether the identified regulatory mechanism applies to other breast cancer subtypes remains to be determined. Several limitations regarding the epigenetic mechanism should be acknowledged. Although ZEB1 overexpression was accompanied by enrichment of H3K27ac and H3K4me3 at the IL-6 promoter, the present study did not examine the direct recruitment of transcriptional co-activators or chromatin-remodeling complexes. Moreover, the increased promoter methylation detected by MSP requires further validation using higher-resolution approaches. Therefore, the current findings establish an association between ZEB1-dependent IL-6 activation and the local chromatin state but do not demonstrate that ZEB1 directly mediates chromatin remodeling.

A recent study in colorectal CSCs reported that IL-6 can induce ZEB1 expression through STAT3-PI3K signaling (29), raising the possibility of reciprocal regulation between ZEB1 and IL-6. However, this potential feedback mechanism was not examined in the present study and requires further investigation.

In conclusion, we demonstrate that ZEB1 directly activates IL-6 transcription and identify IL-6 as an important functional mediator of ZEB1-induced CSC-associated properties in TNBC cells. ZEB1-dependent IL-6 activation was accompanied by enrichment of the active histone marks H3K27ac and H3K4me3 at the IL-6 promoter and was potentiated by AZA/TSA treatment, indicating that this regulation is influenced by the local epigenetic context. These findings extend current understanding of the ZEB1–IL-6 regulatory axis and support further investigation of this axis as a potential therapeutic target in ZEB1-high TNBC.

## Data Availability

The RNA-sequencing data generated in this study have been deposited in the NCBI Gene Expression Omnibus (GEO) under accession number GSE305584.
