# Supplementary material for: ZEB1 directly activates IL-6 to promote triple-negative breast cancer stemness in an epigenetically constrained context
Source: Front Oncol. 2026 Sep 1;16:1929305. doi: 10.3389/fonc.2026.1929305 (PMC13574650; doi:10.3389/fonc.2026.1929305)
Supplement: Supplementary file 1 [file DataSheet1.docx]

**ZEB1 directly activates IL-6 to promote triple-negative breast cancer stemness in an epigenetically constrained context**

Weiyue Zhang et al.

**Supplementary Figure 1.** **ZEB1 is associated with tumor-secreted cytokines and chemokines. A.** KEGG analysis of DEGs of Ctrl/231 and ZEB1/231. **B.** Heatmap of differentially expressed cytokines and chemokines in Ctrl/231 and ZEB1/231. **C-F.** IL-6 mRNA (qPCR; **C, E**) and secreted protein (ELISA; **D, F**) levels in MDA-MB-231 (**C, D**) and SUM-159 (**E, F**) cells upon ZEB1 overexpression, n = 3. **G-J.** IL-6 mRNA (qPCR; **G, I**) and secreted protein (ELISA; **H, J**) levels in MDA-MB-231 (**G, H**) and SUM-159 (**I, J**) cells upon ZEB1 knockdown, n = 3. Data are represented as the mean ± SEM. ***P* < 0. 01, ****P*< 0. 001.

**Supplementary Figure 2. IL-6 promotes the stemness of SUM-159 cells. A, B.** SUM-159 cells were stably transfected with the IL-6 expression plasmid (IL-6/159) or empty vector control (Ctrl/159). The expression of IL-6 was verified by immunoblotting and normalized to the levels of β-Actin. **C-H.** Flow cytometry analysis of the side-population assay (**C, D**), the CD44^+^CD24^−^ population (**E, F**), and the ALDH activity (**G, H**) in Ctrl/159 and IL-6/159 cells. n = 3. Data are represented as the mean ± SEM. **P* < 0. 05, ***P* < 0. 01, ****P* < 0. 001.

**Supplementary Figure 3. IL-6 mediates ZEB1-induced stemness in SUM-159 cells.** **A.** Secretion of IL-6 in Ctrl/159, ZEB1/159, and ZEB1/159 + shIL-6 cells. **B, C.** Western blot analysis and quantification of IL-6. **D-G,** Flow cytometry analysis of the side population (**D, E**) and the CD44^+^CD24^-^ cell population (**F, G**) in the indicated SUM-159 cells. n = 3. Data are represented as the mean ± SEM. **P* < 0. 05, ***P* < 0. 01, ****P* < 0. 001.

**Supplementary Figure 4.** ZEB1 induces an active chromatin state to drive IL-6 transcription. **A, B**. Quantification of ZEB1 (**A**) and IL6 (**B**) protein levels (normalized to GAPDH) from the western blot in Figure 6C. **C-E**. ChIP-qPCR analysis of H3K27ac (**C**), H3K4me3 (**D**), and H3K27me3 (**E**) enrichment at the IL-6 promoter E2-box elements. n = 3. Data are represented as the mean ± SEM. ***P* < 0. 01, ****P* < 0. 001.

**Supplementary Table 1:** Sequences of the oligonucleotides for Overexpression or shRNA, and site-mutagenesis.

| Construction of overexpression vector | |
| --- | --- |
| ZEB1- forward | 5’-GCTCTAGAGCCACCATGGCGGATGGCCCCAGGTGTA-3’ |
| ZEB1- reverse | 5’-CGGGATCCTTAGGCTTCATTTGTCTTTTCTT-3’ |
| IL6- forward | 5’-TCCCCCGGGATGAACTCCTTCTCCACAAGCGCCT-3’ |
| IL6- reverse | 5’-CGGGATCCCTACATTTGCCGAAGAGCCCT-3’ |
| Construction of shRNA vector | |
| shIL6-1 | 5’-GGATGCTTCCAATCTGGAT-3’ |
| shIL6-2 | 5’-GGAAAGTGGCTATGCAGTT-3’ |
| Quantitative PCR | |
| hIL-6-F | AGAGGCACTGGCAGAAAACAAC |
| hIL-6-R | AGGCAAGTCTCCTCATTGAATCC |
| Construction of IL-6 promoter | |
| -1991-forward | 5’-GGGGTACCATGTAACAGCCAGGATCAAAC-3’ |
| -1067-forward | 5’-GGGGTACCGTGCCTGCGTCCGTAGTTT-3’ |
| -721-forward | 5’-GGGGTACCCAGCAGCCAACCTCCTCTAAG-3’ |
| -300-forward | 5’-GGGGTACCCAAGACATGCCAAAGTGCTGA-3’ |
| reverse | 5’-CGACGCGTGGAGGGGAGATAGAGCTTCTC-3’ |
| Methylation specific PCR | |
| m-MSP forward | 5’-GACGGATTATAGTGTACGGTTGC-3’ |
| m-MSP reverse | 5’-ATAAAATCATCCATTCTTCACCG-3’ |
| u-MSP forward | 5’-GATGGATTATAGTGTATGGTTGTGG-3’ |
| u-MSP reverse | 5’-ATAAAATCATCCATTCTTCACCAAT-3’ |
| Site-Directed Mutagenesis of IL-6 promoter | |
| Mut-I forward | 5’-caggatcaaacggttaggaagacgag-3’ |
| Mut-I reverse | 5’-CTCGTCTTCCTAACCGTTTGATCCTG-3’ |
| Mut-Ⅱ forward | 5’-GGGGCCCATGCAGGTGCCCCAGTGAA-3’ |
| Mut-Ⅱ reverse | 5’-TTCACTGGGGGAGCCGCATGGGCCCC-3’ |
| Mut-Ⅲ forward | 5’-tgggctgaagcGgCtCaagaaagtgg-3’ |
| Mut-Ⅲ reverse | 5’-CCACTTTCTTGAGCCGCTTCAGCCCA-3’ |
| Mut-Ⅳ forward | 5’-tcacacactccgcttagagacgcctt-3’ |
| Mut-Ⅳ reverse | 5’-AAGGCGTCTCTAAGCGGAGTGTGTGA-3’ |
| Quantitative ChIP | |
| IL-6-E1-F | GGTCCTTGATGTAACAGCCAG |
| IL-6-E1-R | AAGTGTCTCCTTCCTCTCCTG |
| IL-6-E2-F | AATCAAGACTTACAGGGAGAGG |
| IL-6-E2-R | GAGTCTCTTCACCACTGTTTCAC |
| IL-6-E3-F | CAGTGAAACAGTGGTGAAGAGAC |
| IL-6-E3-R | GACTCCTTTTTGCCACCGC |
| IL-6-E4-F | CGCGGTGGCAAAAAG |
| IL-6-E4-R | TTCTGTGTTCTGGCTCTCC |
